# Green Synthesis of ZnO/SnO_2_ Hybrid Nanocomposite for Degradation of Cationic and Anionic Dyes under Sunlight Radiation

**DOI:** 10.3390/ma16237398

**Published:** 2023-11-28

**Authors:** Naaser A. Y. Abduh, Abdel-Basit Al-Odayni

**Affiliations:** 1 Department of Chemistry, College of Science, King Saud University, Riyadh 11451, Saudi Arabia; 2 Restorative Dental Sciences Department, College of Dentistry, King Saud University, Riyadh 11545, Saudi Arabia

**Keywords:** zinc oxide, tin oxide, nanocomposite, photocatalyst, photodegradation, dyes

## Abstract

The aim of this work was to biosynthesize SnO_2_-decorated ZnO (ZT) nanocomposites (NCs) of different Sn content (10, 20, and 30 mol%), namely, ZT10, ZT20, and ZT30, using *Olea europaea* leaf aqueous extract-based phytocompounds as nanoparticle facilitating agents for application as effective photocatalyst in the removal of dyes from polluted water. The obtained ZT NCs were characterized using various techniques, including FTIR, XRD, TGA, TEM, EDS, UV–Vis, PL, and BET surface area. X-ray diffraction patterns show that rutile SnO_2_ and hexagonal ZnO coexist in the composites, and their crystallite size (D) is affected by the SnO_2_ ratio; the obtained D-values were 17.24, 19.07, 13.99, 6.45, and 12.30 nm for ZnO, SnO_2_, ZT10, ZT20, and ZT30, respectively. The direct band gaps of the ZT heterostructure increase with increasing SnO_2_ ratio (band gap = 3.10, 3.45, 3.14, 3.17, and 3.21 eV, respectively). TEM spectroscopy revealed nanorod and spherical grain morphologies of the composites, while EDS confirmed the elemental composition, the element ratio, and the composite’s purity. All catalysts exhibit type III isotherm with macropore structure. The photocatalytic efficiency against cationic (methylene blue (MB), rhodamine B (RB)), and anionic (methyl orange (MO)) dyes, under sunlight, was optimal with ZT20. The results revealed almost complete degradation at 55, 65, and 55 min, respectively. Hence, it is evident that incorporating SnO_2_ improves the photocatalyst’s performance, with an apparent optimal enhancement at 20 mol% Sn decorating ZT NCs. More interestingly, the catalyst stability and activity remained unaffected even after four activating cycles.

## 1. Introduction

Water is an essential resource for human life and development, making its pollution a concern to humanity [1]. The current accelerated environmental changes and water contamination have enforced a scientific direction to find answers and lessen pollution. Among many pollutant foundations, organic dyes are dominant due to their stability, causing adverse effects on humans and living organisms [2]. The use of dyes is ubiquitous, whether it is in textiles, foods, cosmetics, medicines, or other products. The textile sector, in particular, significantly impacts the amount of dye released into the environment [3,4]. Therefore, treatments and taking the necessary measures to address these problems have become urgent and essential and have fallen upon the scientific community. As a result, multiple physical, chemical, biological, and combinatorial techniques have emerged to treat water and mitigate the effects of dye pollution [5,6,7].

Advanced oxidation processes, photocatalytic degradation in particular, have emerged as a promising, reliable, and safer technique that can be an alternative to traditional methods such as adsorption and coagulation [8]. Photocatalysis is the process by which light changes the rate of chemical reactions in which UV radiation, visible radiation, or an ultraviolet and visible combination can be used [9]. However, direct solar radiation is economically and environmentally preferable [10]. The economic and environmental importance of photocatalysis has increased dramatically over the past decade because of its advantages by performing the oxidation of a wide range of synthetic dyes, complete mineralization to carbon dioxide and water, using solar light, which has low cost, low energy consumption, is non-toxic, and has high efficiency. In addition, the simplicity of the reaction conditions, such as room temperature and normal atmospheric pressure, enhances its merit [11,12].

Due to their properties, such as light absorption, metal oxides and their composites are successful photocatalysts for degrading organic pollutants [13]. Nonporous metal oxides’ surface, structure, and crystalline properties make them suitable for photocatalytic applications in water treatment [14]. Moreover, an ideal semiconductor photocatalyst should be advanced with an appropriate band gap, non-toxicity, high water stability, abundance on Earth, and cost-effectiveness [15]. Titanium dioxide (TiO_2_), zinc oxide (ZnO), tin oxide (SnO_2_), vanadium pentoxide, tungsten trioxide, copper(I) oxide, bismuth(III) oxide, etc., have been widely reported as photocatalysts [16]. However, TiO_2_ and ZnO are two protuberant examples of stable, cost-effective, efficient, and environment-friendly photocatalysts for dye degradation in water [17]. As a photocatalyst, ZnO is dominant in the dye degradation field under sunlight due to its band energy of 3.2 eV [18]. However, the practical application of ZnO is still limited by the low photocatalytic efficiency caused mainly by electron-hole recombination [19]. Thus, metal oxide nanocomposites with other oxides can ensure greater photocatalytic efficiency and lower electron-hole recombination than pure oxides [20]. SnO_2_ is a better electron acceptor due to its more positive conduction band edge, making it an excellent option for the heterostructure component [21]. For this reason, ZnO and SnO_2_ nanocomposites are of interest to researchers in water treatment [22,23]. For example, Binghua Yao et al. have used a series of ZnO-SnO_2_ composites for methylene blue (MB) degradation under mercury lamp irradiation. They mentioned that the heterojunction effect enhanced the activity of the composite toward dye degradation compared to ZnO and SnO_2_ alone [24].

Many methods, such as co-precipitation [25], sol–gel [26], and hydrothermal [27], were used to prepare the ZnO-SnO_2_ heterostructure. Remarkably, the green synthesis method has made strong inroads as an environmentally promising method that possesses several advantages that make it attractive to several researchers [28]. Flower extract was employed as a natural reducing and stabilizing agent to synthesize ZnO-SnO_2_ nanocomposite as a photocatalyst for Eriochrome black T dye degradation under sunlight irradiation. Particle sizes ranging from 5 to 40 nm with an average of 22 nm were obtained, indicating a good performance of the green synthesis route [29]. ZnO-SnO_2_ can be an efficient photocatalyst under sunlight, visible light, and UV. Thus, the ZnO-SnO_2_ photocatalyst was tested by Hamrouni et al. for MB degradation using different light sources [30]. A dependence between the degradation rate and the light source was found, and solar light showed higher activity than artificial visible light. Based on the above, the number of reports in which ZnO-SnO_2_ was synthesized by the green method and applied to degrading dyes under sunlight needs to be enriched.

Herein, this work aims to synthesize ZnO-SnO_2_ nanocomposite using *Olea europaea* leaf plant extract as a simple, green, and cost-effective approach. FRIR, XRD, EDS, TEM, TGA, BET, UV, and PL analysis were used for complete characterization. Then, the as-prepared photocatalyst was tested for cationic dyes (MB, RB) and anionic dyes (MO) degradation under direct sunlight irradiation. Kinetic studies were also performed.

## 2. Materials and Methods

### 2.1. Materials and Reagents

*Olea europaea* leaves were collected from Asir Province, Abha region, Saudi Arabia. The chemicals were used as provided without further purification and are as follows: ammonium hydroxide (NH_4_OH, 25% in water) (Sigma-Aldrich, St, Louis, MO, USA); tin (IV) chloride pentahydrate (SnCl_4_·5H_2_O) (Sigma-Aldrich, USA); zinc acetate dihydrate (Zn(CH_3_COO)_2_·2H_2_O) (BDH Chemicals, Poole, UK); methyl orange (British Drug Houses Ltd. (BDH) Chemicals Ltd., UK); methylene blue (Sigma-Aldrich, USA); rhodamine B (Sigma-Aldrich, USA).

### 2.2. Preparation of Catalysts

Preparation of aqueous extract. *Olea europaea* leaves were washed with deionized water and dried in the shade for two weeks. Then, 10 g of the dry leaves was crushed into small pieces, immersed in 100 mL deionized water, and boiled at 55 °C for 20 min. The resulting solution was filtered using filter paper and stored at 4 °C.

Preparation of ZnO and SnO_2_ NPs. A total of 0.015 moles of zinc acetate dihydrate or tin (IV) chloride pentahydrate was added to 30 mL of extract and stirred for 1 h, after which an appropriate amount of precipitating agent (NH_4_OH, 2.5 M) was added dropwise. Upon completion of prepetition, the temperature was raised to 60 °C with stirring in a closed vessel for 3 h. The precipitate was left for 8 h for aging, then filtered, washed several times with aqueous ethanol solution (1/3 *vol*/*vol*), and dried in an oven at 75 °C overnight. Calcination was performed in an oven at 550 °C for 6 h under air to obtain the individuals’ ZnO and SnO_2_. Using the same condition, the composites (Sn/Zn mole ratio = 10%, 20%, and 30%) were prepared by adding both zinc and tin precursor simultaneously to the extract.

### 2.3. Photocatalytic Dye Degradation

Typically, 10 mL of the dye solution (10 ppm) and 10 mg of the photocatalyst were mixed in a closed cylindrical bottle, representing the photocatalytic system. To achieve the adsorption/desorption equilibrium, the resulting suspension was magnetically stirred in the dark for 30 min. Furthermore, a blank solution without a catalyst was set out under the same conditions. The solution was then exposed to radiation in the open air under direct sunlight at atmospheric pressure. Samples from the catalytic system (the suspension) were taken out at certain intervals, centrifuged to separate the catalyst, and then measured for the remaining dye concentration using UV-Vis spectrophotometer. Absorption intensity was used at λmax = 664, 554, and 462 nm for MB, RB, and MO, respectively. The concentration was calculated based on calibration curves established for each dye according to Beer–Lambert Law, Equation (1). The photodegradation ratio is measured as given by Equation (2).

(1) A=εbc

(2) $$Degradation={(C}_{0}-C_{t})/C_{0}$$

where *A* is the absorbance, *ε* is the absorptivity, *c* is the concentration, *C*_0_ and *C_t_* are the dye concentration at initial and at time *t* (min) of the process.

### 2.4. Characterization

To obtain Fourier transform infrared (FTIR), a solid sample of about 2–3 mg was combined with about 0.5–1 g KBr, ground in a mortar to a fine, well-mixed powder, and then formed into a disk by exerting high pressure on a Perkin Elmer Spectrum BX (Perkin Elmer, Waltham, MA, USA). The infrared frequency is between 400 and 4000 cm^−1^. The Rigaku XtaLAB small II benchtop X-Ray 110 crystallography machine (The Woodlands, TX, USA) was used to perform the X-ray diffraction (XRD) measurements. Cu Kα radiation, λ = 1.5418 Å was employed as a light source. The powdered sample was put in a sample container and subjected to X-ray radiation at room temperature. The pattern was recorded in the range of 2 theta between 10 and 80 degrees and at a rate of 3 degrees per minute (Micromeritics Instrument Corporation, Norcross, GA, USA). USA’s Micromeritics Tristar II 3020 surface area and porosity analyzer was used to assess the catalyst’s specific surface area, pore volume, and average pore diameter. Analysis was run on the sample to determine the BET surface area using a computer connected to the equipment. UV-Vis analysis was performed using a PDA UV/Vis Lambda 265 Spectrophotometer (PerkinElmer, Waltham, MA, USA). The photoluminescence (PL)spectra were measured using Hitachi F-4600 (Hitachi, Tokyo, Japan). The excitation wavelength was 320 nm, and fluorescence spectra were recorded between 330 and 600 nm. Transmission electron microscopy (TEM) was performed using a JEM-2100F field emission electron microscope from JEOL (Tokyo, Japan) with an 80 kV acceleration voltage. A drop in the sample’s colloidal suspension was placed on a copper grid of lacey carbon for TEM characterization. Thermogravimetric analysis (TGA) of catalysts was carried out on a Mettler Toledo TGA/DSC Star system (Columbus, OH, USA) in a nitrogen environment at a heating rate of 10 °C min^−1^. An X-MaxN system from Oxford Instruments (Abingdon, UK) was used to obtain an energy-dispersive X-ray spectroscopy (EDS) profile.

## 3. Results and Discussion

### 3.1. Catalyst Characterizations

#### 3.1.1. FTIR Analysis

**Figure 1:**
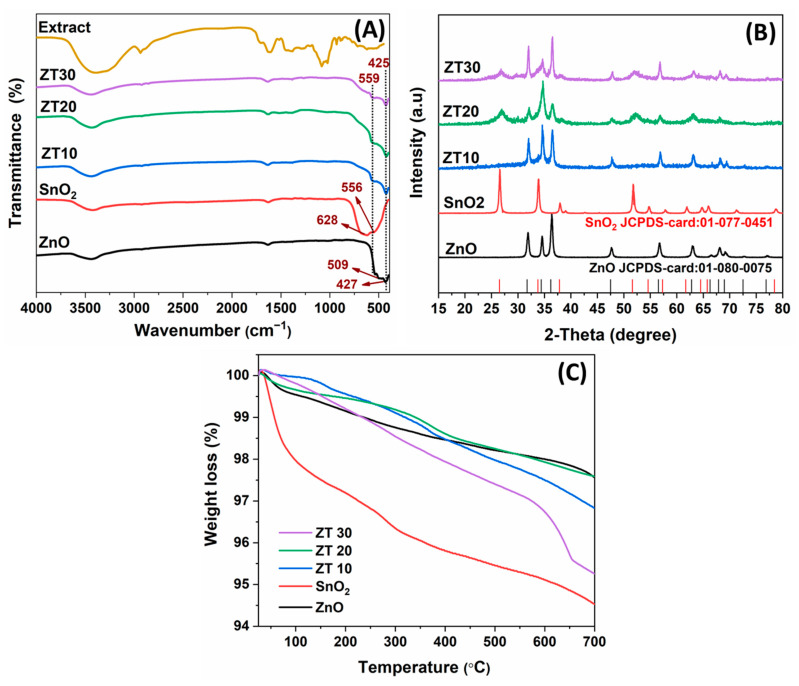
(**A**) FTIR spectra. (**B**) XRD diffractograms. (**C**) TGA thermograms of ZnO, SnO_2_, ZT10, ZT20, and ZT30.

The FTIR analysis of the extract, SnO_2_, ZnO, Z10, ZT20, and ZT30 NPs is explored in Figure 1A. The absorption of SnO_2_ NPs is typically seen at 628 and 556 cm^−1^ and assigned to O-Sn-O and Sn-O stretching vibration modes, respectively [31]. The characteristic peaks of ZnO are located at 509 and 427 cm^−1^ [32]. The prepared three composites showed two prominent peaks at 559 and 425 cm^−1^, which confirm the presence of SnO_2_ and ZnO in the composite. Moreover, water adsorbed on the surface appears at 3450 and 1628 cm^−1^. The IR spectrum of the extract was also visualized. The spectrum is typical of extracts, showing multiple peaks for the corresponding capping and stabilizing organic compounds that contribute to the production of NPs. Thus, the broad peak ranging from 3436 to 3249 cm^−1^ can be assigned to the O-H group of water, phenolic compounds, and different types of amine N-H functionalities. The peaks around 2930 and 2889 cm^−1^ are assignable to the C–H stretching of aliphatic compounds, while the peaks centered at 1619 cm^−1^ are for unsaturated C=C groups. The carbonyl functional group was also confirmed at 1704 and 1636 cm^−1^, while the bands at 1380 and 1021 cm^−1^ belong to the stretching C–O bond.

#### 3.1.2. XRD Analysis

The XRD patterns for the as-prepared photocatalysts are shown in Figure 1B. The SnO_2_ phase prominent peaks are located at 2θ values of 26.60°, 33.83°, 37.89°, and 51.78°, which are associated with (110), (101), (200), and (211) miller indices. Likewise, the characteristic diffraction peaks of ZnO are seen at 31.82°, 34.60°, 36.28°, 47.72°, 56.67°, and 63.07° corresponding to (100), (002), (101), (102), (110), and (103) planes [33]. Moreover, hexagonal wurtzite ZnO and tetragonal SnO_2_ have a well-defined diffraction peak consistent with JCPDS references [01-080-0075], indicating well-crystalline structures. More importantly, compared to ZnO diffraction peaks, the XRD patterns of ZT20 and ZT30 composites revealed additional peaks at 2*θ* values of 26.81°, 37.86°, and 51.92° corresponding to the pure SnO_2_. The diffractograms also showed no other characteristic peaks, indicating the purity of the prepared ZT composites. It is evident that the ZnO degree of crystallinity is deceased with increasing SnO_2_ content, a finding that aligns with previous observations [26,34]. By increasing SnO_2_ content, the intensity of its corresponding peaks increased, more clearly for composites ZT20 and ZT30 compared to ZT10; however, the ZT10 diffractogram revealed no apparent change compared to ZnO pattern due to either the composite’s lower SnO_2_ content or the very low crystallinity of SnO_2_ [26]. These findings are consistent with other published reports [35,36]. In addition, it is evinced that overlapping of peaks from both oxides leads to peak broadening. Thus, intensity is reduced accordingly, as seen for the peak at 34.77°, which is the center for overlapped peaks assigned at 34.60 and 33.80 belonging to ZnO and SnO_2_ phases, respectively. A similar observation can be detected in the literature [30,34].

The crystallite size and distributions of ZnO, SnO_2_, ZT10, ZT20, and ZT30 were calculated based on the Debye–Scherrer equation, Equation (3).

(3) $$D=\frac{K\lambda }{\beta cos\theta }$$

where *D* is the crystallite size; *λ* is the wavelength of the X-ray beam; *K* is the Scherrer constant; *θ* is a Bragg angle; and *β* is the full width at half maximum (FWHM) of the diffraction peak.

The particle size of the high-intensity peaks of the as-prepared samples were extracted and displayed in Table 1. The results indicated higher crystallite size for individual oxides ZnO (17.27 nm) and SnO_2_ (19.07 nm) than the corresponding composites (ZT10, ZT20, and ZT30). Notably, the D-value of ZT20 was the lowest (6.45 nm) compared to ZT10 (13.99 nm) and ZT30 (12.30). Moreover, the low D-values of composites compared to pure oxides could confirm the inhibitory effect of SnO_2_ on the growth of ZnO grains. However, the discrepancy in the D-value presented in Table 1 could be explained in terms of the imperfections [37]. Hence, the addition of SnO_2_ continuously reduces the growth rate of ZnO crystallites from 17.24 to 13.99 (ZT10) and 6.45 (ZT20); then, by increasing the SnO_2_ content to 20 mol% in ZT20, the D-value increased. This behavior is a result of increased imperfections in the crystal structure, such as the point of defects and dislocations, which restrict the growth of crystallites and grains according to the Zene pinning effect [38], but the higher Sn content may lead to its own phase structure, thus reducing its influence on the formation of other crystals [37,38].

#### 3.1.3. TGA Analysis

The thermal stability of the catalysts was performed using the TGA technique, and the resulting thermograms are illustrated in Figure 1C. As can be seen, the catalysts under investigation have high thermal stability, with about 0.5 wt% mass loss of up to 200 °C. However, a weight loss of about 2.3 wt% was observed at up to 150 °C for SnO_2_, which could be attributed to the moisture content in the sample. In addition, two weight loss phases (1.82 wt% and 1.3 wt%) were detected in the temperature range from 150 to 400 °C and from 400 to 700 °C. The first stage is for the physically bound moister content, and the second is for the chemically bound water [39].

#### 3.1.4. BET-Surface Analysis

**Figure 2:**
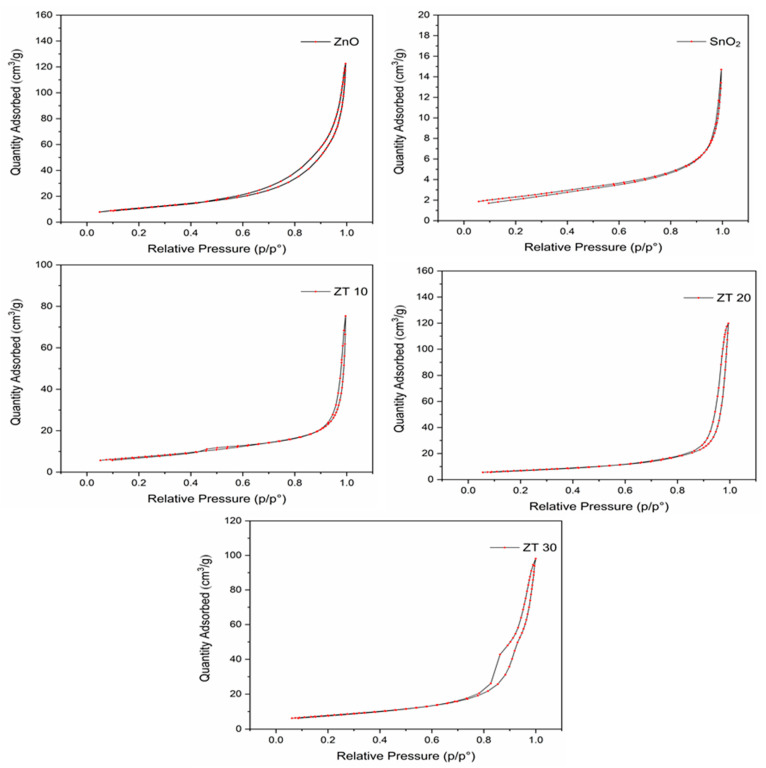
BET surface area plot of ZnO, SnO_2_, ZT10, ZT20, and ZT30.

Using N_2_ adsorption–desorption isotherms and the Barrett–Joyner–Halenda (BJH) method, the BET-surface area, pore volume, and pore width of the investigated photocatalysts have been measured, and the results are summarized in Table 2. According to the IUPAC classification, all photocatalysts are type III isotherms. In addition, Figure 2 confirms the macropore structure of synthesized photocatalysts as a hysteresis loop set at a relative pressure (P/P^o^) close to unity [40]. Furthermore, the surface area of pristine oxides was in the range terminals, with ZnO having the highest surface area of 38.5387 (m^2^/g) and SnO_2_ having the lowest (7.9516 (m^2^/g)). The composite’s surface area, on the other hand, is in between and is closer to the ZnO values. As can be seen from Table 2, the BET specific surface area (SSA) of the composites ZT10, ZT20, and ZT30 are 24.1879, 25.3492, and 27.1261 m^2^/g, a slight increase as the SnO_2_ content increased. This illustrates, however, that the final SSA depends not only on the contribution of each metal oxide in the composites but also on several additional variables, including aggregation, grain size, pore type, shape geometry, and distribution [41].

#### 3.1.5. TEM Analysis

**Figure 3:**
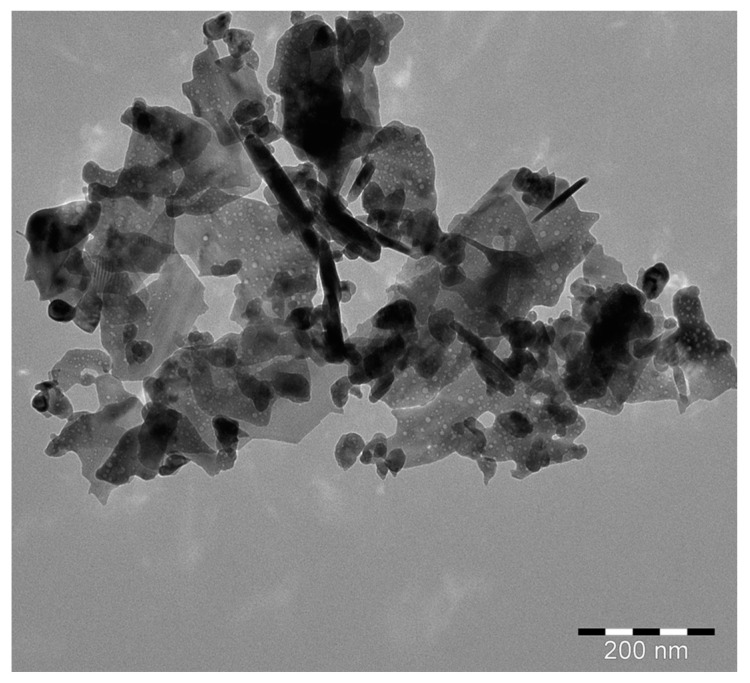
TEM micrograph of ZT20 catalyst.

Transmission electron microscope (TEM) analysis of the catalysts was used to evaluate the surface morphology, particle shape, and particle size. The TEM micrograph of the ZT20 photocatalyst is given in Figure 3. As can be seen, the particles are primarily nanorod structures, with a length of 40–130 nm and a diameter of 10–20 nm. However, additional particles with spherical shapes and about 5–15 nm in diameter were also seen.

#### 3.1.6. EDS Analysis

**Figure 4:**
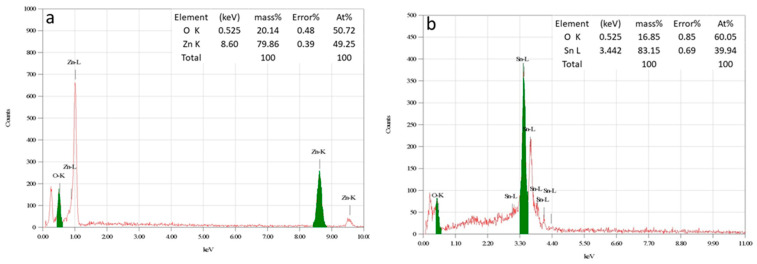

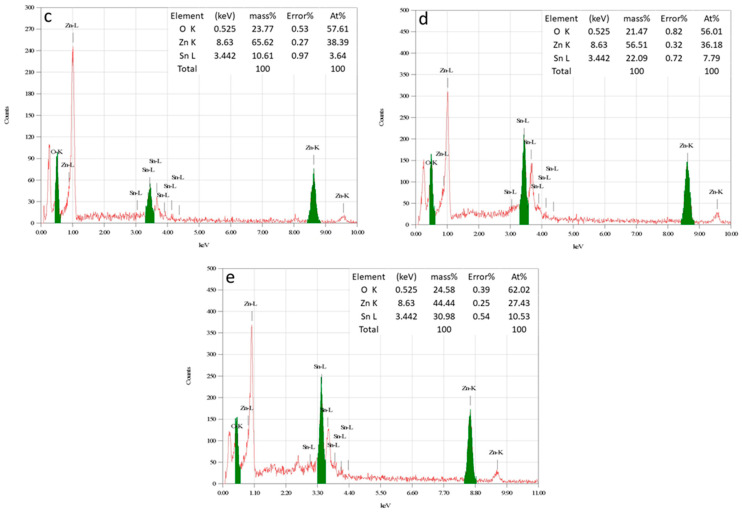
EDS results of (**a**) ZnO, (**b**) SnO_2_, (**c**) ZT10, (**d**) ZT20, and (**e**) ZT30.

The chemical composition of the prepared catalysts was investigated by the EDS technique (Figure 4). The patterns demonstrated the presence of zinc, tin, and oxygen elements in proportions consistent with the values calculated upon preparation. Hence, the resulting experimental mol ratios (%) of Sn in the ZT10, ZT20, and ZT30 composites, as calculated by Equation (4), were 8.66, 17.71, and 27.73 mol%, respectively. Taking into consideration the EDS accuracy, which is relatively low compared to other elemental analysis techniques, the resulting experimental values of Sn At.%, compared to its theoretical values, are acceptable for this technique [42].

(4) $$Sn\;\left(mol\%\right)=\left(\frac{Sn}{Sn+Zn}\right)\times 100$$

#### 3.1.7. UV-Visible Analysis

**Figure 5:**
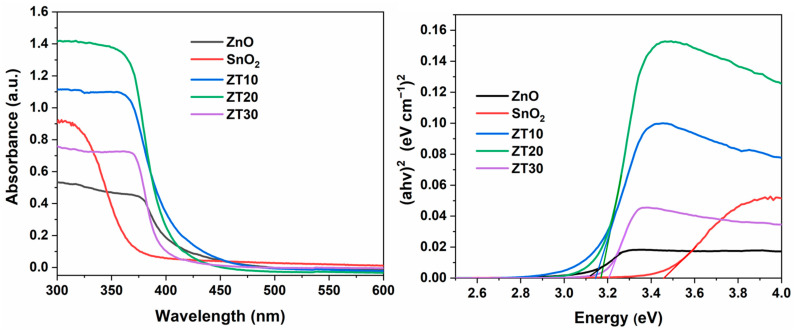
UV-Vis spectra and band-gap plot of ZnO, SnO_2_, ZT10, ZT20, and ZT30.

The UV-Vis spectroscopy and Tauc plot of the synthetic photocatalysts were employed to evaluate the optical band gap of the NPs. Figure 5 represents the UV-Vis spectra and Tauc plot for the band-gap energy calculation for all samples, showing a well-defined absorption feature and *Eg*. The Tauc model equation for calculating the band-gap energy is given in Equation (5).

(5) $$\left(\alpha h\nu \right)=A{\left(h\nu -Eg\right)}^{n}$$

where *h* is Planck’s constant; *α* is the absorption coefficient; *υ* is the photon frequency; *A* is a constant, and *Eg* is the band-gap energy. The value of *n* indicates the nature of the electronic transfer, where *n* = 1/2 or 2 for direct and indirect transfers, respectively. Bulk ZnO and SnO_2_ commonly present a direct energy gap of 3.2 and 3.6 eV, respectively. The band gaps, as calculated by the Tauc equation, fall in the range of 3.10–3.45 eV (Table 1). It can be seen that the band gap increases in line with the SnO_2_ ratio increase, a result that agrees with the published reports [30,43].

#### 3.1.8. PL Analysis

**Figure 6:**
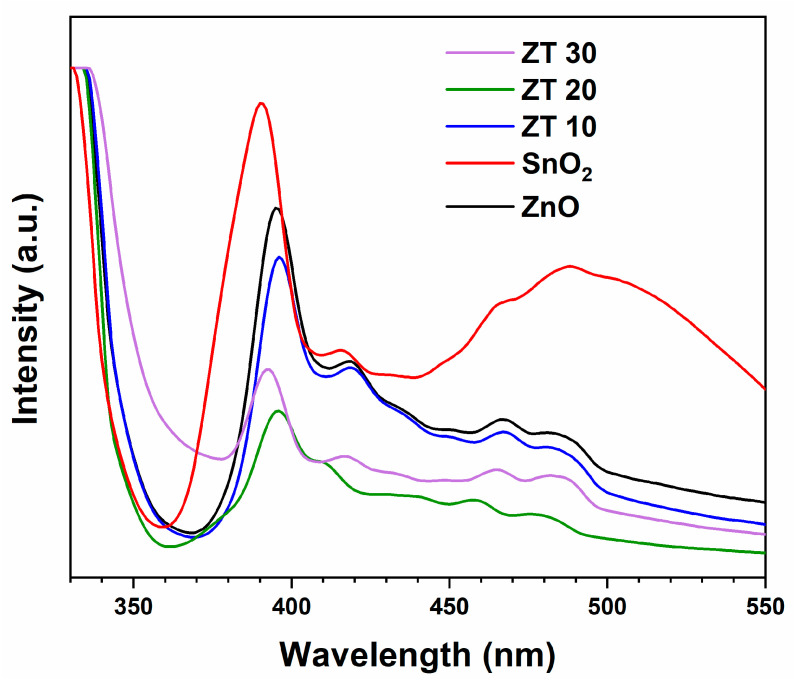
Photoluminescence spectra of ZnO, SnO_2_, ZT10, ZT20, and ZT30.

PL spectra at room temperature and 320 nm as the excitation wavelength were recorded to investigate the optical characteristics of the metal oxides under investigation. Photoluminescence of the substrate is induced by radiative recombination of the photogenerated electron-hole pair. Information about defects, dangling bonds, and vacancies in oxygen ions can be attained from the PL spectra. Most importantly, the electron-hole recombination rate can be obtained, which is crucial in the photocatalyst. From Figure 6, the UV emission peak at 390 nm, which is seen in all photocatalyst spectra, is assigned to the direct electronic recombination between conduction band electrons and holes in the valence band [44,45]. The PL intensity of the prepared nanocomposite was reduced by coupling with SnO_2_, and ZT20 showed a lower recombination rate.

### 3.2. Catalytic Activity

**Figure 7:**
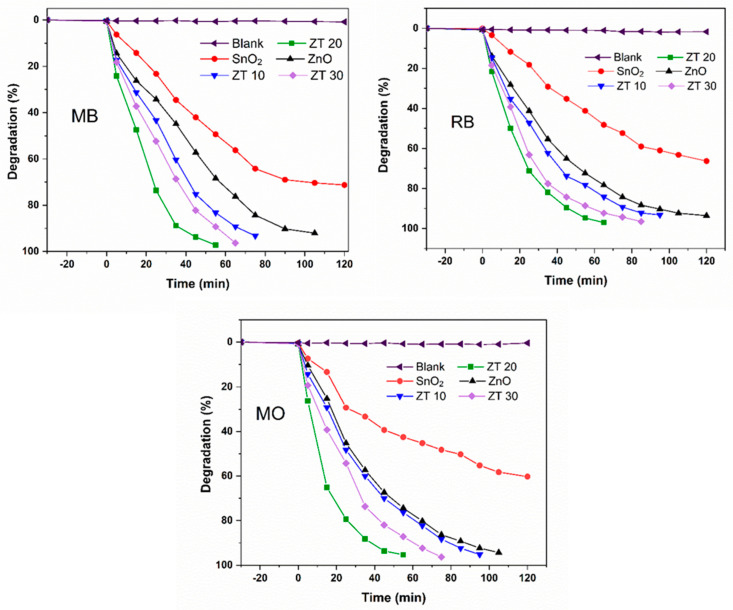
Degradation efficiency of the ZnO, SnO_2_, ZT10, ZT20, and ZT30 for MB, MO, and RB dyes.

To investigate the photocatalytic performance of the prepared nanomaterials, photodegradation of methylene blue (MB), rhodamine B (RB) as cationic dyes, and methyl orange as anionic dye was performed under sunlight irradiation on a clear day. Furthermore, a similar set of conditions was applied to all photocatalytic experiments. A comparison of the photocatalytic performance of ZnO, SnO_2_, ZT10, ZT20, and ZT30 is shown in Figure 7.

There is no significant degradation observed without a catalyst. In contrast, the color of the solution gradually changed over time in the presence of photocatalysts, which indicates the decomposition of colored dye formulations. Since SnO_2_ has a wide band gap, only the ultraviolet radiation in solar radiation can photoexcite it, resulting in a decrease in its activity. On the other hand, ZnO, with a lower band gap, shows higher activity under sunlight than pure SnO_2_. More importantly, the addition of SnO_2_ leads to improved catalytic activity of ZnO. ZT20 showed higher photodegradation efficiency than ZT10 and ZT30.

**Figure 8:**
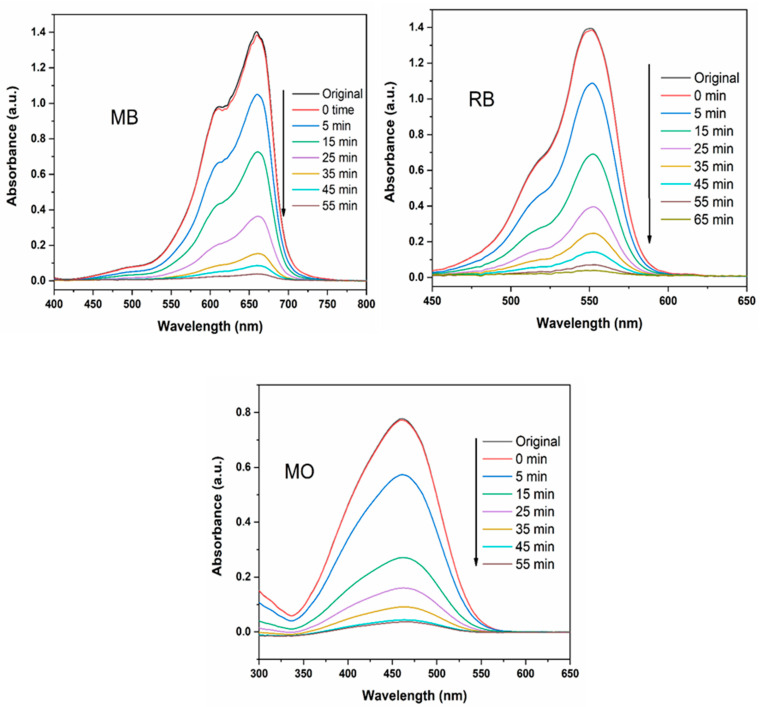
UV–Vis spectra of the MB, MO, and RB dyes at different time intervals under sunlight irradiation using ZT20 photocatalyst.

As shown in Figure 8, the maximum absorption peaks of MB, RB, and MO gradually diminished and almost disappeared under sunlight irradiation during 55, 65, and 55 min, respectively, in the presence of ZT20 nanomaterials.

Photodegradation kinetics followed the Langmuir–Hinshelwood (L–H) model, where the reaction rate depends on the dye concentration, according to Equation (6).

(6) $$ln\frac{C_{o}}{C_{t}}=k_{app}t$$

where *C_t_* and *C*_0_ are the concentrations at the given and initial time (*t*), while *k_app_* represents the apparent rate constant (min^−1^).

**Figure 9:**
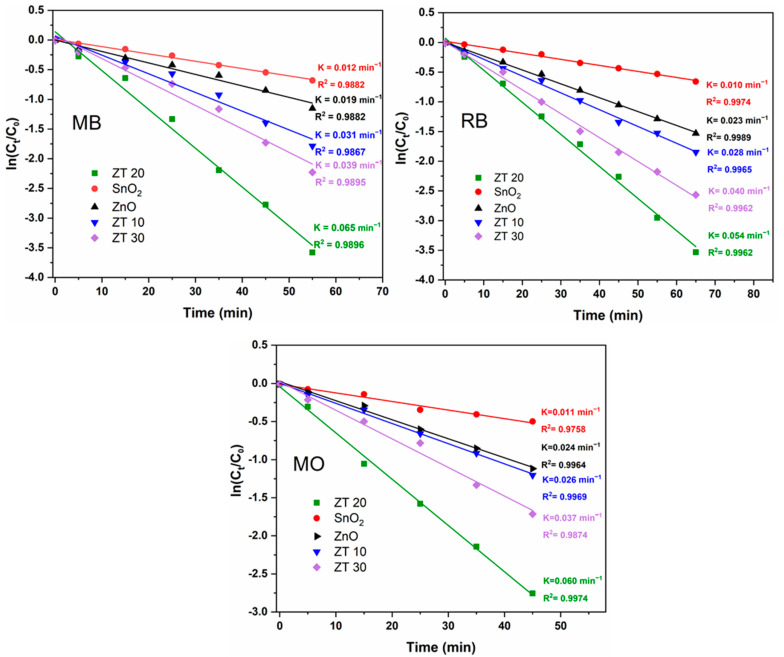
Variations in *ln*(*C_t_*/*C*0) with an irradiation time of the ZnO, SnO_2_, ZT10, ZT20, and ZT30 for MB, MO, and RB dyes.

Kinetic results aligned well with pseudo-first-order kinetics, as seen in Figure 9, where plots of *ln*(*C*_t_/*C_0_*) vs. time showed a linear relationship. When degrading MB, RB, and MO, the rate constant of pure SnO_2_ photocatalyst was much lower than that of the pure ZnO and heterostructure catalyst. Similarly, the pure ZnO showed lesser activity than those with SnO_2_ composite. These findings suggest that the ZnO’s deficient photocatalytic degradation activity under sunlight may have been caused by the high rate of photogenerated charge carrier recombination. Thus, this rate is reduced by incorporating tin oxide, resulting in increased activity.

### 3.3. Reusability

**Figure 10:**
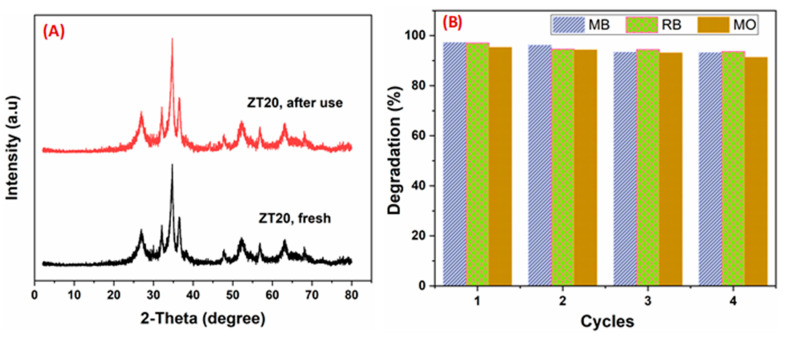
(**A**) XRD patterns for fresh and reused ZT20 photocatalysts. (**B**) Its recycling efficiency (4 cycles) on dyes (MB, RB, and MO) degradation.

Along with activity, reusability is regarded as the most significant indicator in the research of industrial catalysts. Catalysts must be easily removed from and recycled after catalytic reactions, especially for industrial applications, to reduce production costs and limit waste creation. Centrifugation, washing with aqueous ethanol (30% *v*/*v*), and drying at 80 °C for 12 h were all used to recycle the catalyst. Figure 10A depicts the outcomes of an investigation of the catalyst’s stability using the XRD technique. The spectrum is unaltered before and after use, demonstrating the catalyst’s stability. The efficiency of the recycled photocatalyst was examined through four cycles, and the results are shown in Figure 10B. Accordingly, good stability can be observed with no significant decrease in the catalytic efficiency after four cycles.

### 3.4. Relative Catalytic Performance of ZT

The catalytic performance of ZT compared to similar catalysts reported in the literature is summarized in Table 3. To simplify comparison, the catalyst composition, the dye model pollutant, irradiation source, degradation time at almost complete degradation (otherwise, the degradation degree is stated), and normalized efficiency in terms of catalyst mass per degraded dye mass (g/g) were tabulated. As can be seen, the degradation efficiency of the present catalyst ZT20 is well positioned in the list or even superior to the other reported ones. Such performance could be expressed in terms of the fast action (almost complete degradation at 55–65 min), the activity against a broad spectrum of cationic and anionic dyes (e.g., MB, MO, and RB), the use of sustainable and cheap activating source (sunlight), and the displayed high efficiency (100 g/g; catalyst/dye). Indeed, assessment is influenced by the condition variation; the performance is a synthetic method- and concentration-related property; however, for simplicity, efficacy can be compared based on the exposure time for complete degradation [46]. Hence, the activity of ZT20 photocatalyst against dye pollutants is high and performs in a short period, ca. 55 min. Moreover, the green and cost-benefit, represented by the simple synthesis of the catalyst, the green method, and the use of uncostly sunlight, is an additional feature that should be considered.

## 4. Conclusions

In this study, SnO_2_-decorated ZnO (ZT) NCs were biosynthesized facilely using *Olea europaea* leaf extract for photocatalytic degradation of the toxic dyes, namely, methylene blue (MB), methyl orange (MO), and rhodamine blue (RB) in polluted water. The catalyst properties were proven using various techniques, including FTIR, UV-Vis, PL, XRD, TEM, EDS, and BET-SSA. The photocatalytic activity depended on the Sn/Zn mol ratio, which is optimal at Sn 20 At.% (ZT20) and exceeded that for individual oxides, ZnO and SnO_2_. The catalytic performance against cationic (MB and RB) and anionic (MO) dyes in an aqueous solution under direct sunlight and ambient atmospheric pressure was effective, and the dyes were almost totally degraded after 65 min. The stability assessment indicated perfect functioning even after four cycles. These findings support the potential use of the investigated composites for environmental remediation by catalytically removing different dyes from contaminated water under solar radiation and atmospheric conditions.

## Figures and Tables

**Table 1:** Crystallite size of the as-prepared photocatalysts individuals (ZnO and SnO_2_) and composites (ZT10, ZT20, and ZT30).

Catalyst	Crystalline Size (nm)	Band Gap (eV)
ZnO	17.24	3.10
SnO_2_	19.07	3.45
ZT10	13.99	3.14
ZT20	6.45	3.17
ZT30	12.30	3.21

**Table 2:** BET surface area and porosity results for the studied catalysts.

Catalyst	Surface Area (m^2^/g)	Pore Volume (cm³/g)	Pore Size (Å)
ZnO	38.5387	0.179606	168.217
SnO_2_	7.9516	0.019655	123.150
ZT10	24.1879	0.175623	312.886
ZT20	25.3492	0.096356	170.283
ZT30	27.1261	0.143314	209.602

**Table 3:** Comparison of the catalytic performance of ZT with alike catalysts reported in the literature.

Composition (Sn/Zn, mol)	D	Dye Pollutant	Light Source	Degradation Time (~100%)	Efficiency (Catalyst/Dye; g/g)	Ref.
0.05:1		MB	Ultraviolet	90 min	16	[30]
			Visible	240 min (70%)		
			Solar	240 min (75%)		
2:1		MO	Ultraviolet	30 min	125	[47]
1:1	16.5	MO	Ultraviolet	60 min (42%)	50	[48]
		MB		15 min (97%)		
1:0.03		RB	Ultraviolet	240 min	100	[49]
			Visible	13.3 hrs		
1:2		MO	Ultraviolet	60 min	125	[50]
1:4	6.5	MB	Solar	55 min	100	This work
		MO		55 min		
		RB		65 min		

Abbreviation: MB = methylene blue; MO = methyl orange; RB = rhodamine blue.

## Data Availability

The data that support the findings of this study are reported in the manuscript.
